# The effects of diet-induced obesity on glucose metabolism in the mouse endometrium

**DOI:** 10.1186/s44494-026-00008-8

**Published:** 2026-11-04

**Authors:** Sameeksha Bhaye, Kassandra Sandoval, Chanaka Rabel, Malia D. Berg, Bruce R. Southey, Kelly S. Swanson, Romana A. Nowak, Matthew Dean

**Affiliations:** 1 Animal Science Laboratory, Department of Animal Sciences, University of Illinois Urbana-Champaign, Urbana, IL 61801 USA; 2 Division of Nutritional Sciences, University of Illinois Urbana-Champaign, Urbana, IL 61801 USA

**Keywords:** DIO models, Endometrium, Glucose metabolism, Insulin signaling

## Abstract

**Background:**

Embryo transfer studies in women with high BMI show lower implantation rates, indicating impaired uterine function. Most mouse studies using diet-induced obesity (DIO) do not characterize the metabolomic phenotype in their models. Additionally, studies usually focus on implantation sites, ignoring the role of the epithelium in supporting embryo development. At proestrus, the uterine epithelium metabolizes glucose for its own needs and stores glycogen to support embryo development after mating. Our objectives were to (1) thoroughly characterize systemic metabolism in a long-term DIO mouse model, and (2) determine how DIO affects glucose metabolism in the uterine epithelium at proestrus.

**Methods and results:**

Mice were randomly assigned to one of three experimental diets (10%, 45%, or 60% kcal from fat) and fed ad libitum. Both high-fat diet (HFD) groups progressively increased in body mass and glucose intolerance during the course of the experiment. After 18 weeks, the mice were sacrificed at proestrus. In serum, gas chromatography-mass spectrometry (GC–MS) showed that HFDs altered the levels of 23 metabolites (*P*<0.05). Analysis of these metabolites showed enrichment in pathways related to lipids, glucose, and energy metabolism. Confirming that the HFD mice were insulin-resistant, liver glycogen stores decreased, while hepatic triglyceride levels were increased in the 60% fat group, in agreement with observations in obese people. In contrast, effects on the uterus were minimal. There was no difference in immunostaining in the uterus for the glycolytic enzymes PKM2 and PFKM. There was no difference in the glycogen levels or immunostaining for glycogen-metabolizing enzymes in the endometrium. Additionally, immunostaining for phospho-IRS and phospho-AKT in the uterine epithelium was similar across groups, indicating no change in AKT signaling in fasted mice. GC–MS analysis of the uterus found alterations in only 10 metabolites in the 60% fat group relative to the 10% fat group (*P*<0.05). Analysis of those metabolites found 4 pathways to be significantly enriched—glycosaminoglycan biosynthesis, mannose type O-glycan biosynthesis, linoleic acid metabolism, and purine metabolism.

**Conclusion:**

Our DIO model resulted in systemic traits indicative of insulin resistance. However, obesity had a minimal effect on glucose metabolism in the endometrium at proestrus.

**Supplementary Information:**

The online version contains supplementary material available at https://doi.org/10.1186/s44494-026-00008-8.

## Introduction

Obesity (body mass index [BMI] > 30) is a rising concern worldwide. As of 2018, 42% of people in the U.S. were obese, and by 2030, 1 in 2 adults will be obese, according to the Centers for Disease Control and Prevention (CDC) [1]. People with obesity are at risk of metabolic dysfunction and reduced fertility [2]. In women, obesity is associated with menstrual dysfunction, anovulation, infertility, miscarriage, and pregnancy complications [3]. Numerous studies with large cohorts of female participants have shown a negative association between obesity and reproductive outcomes [4–6]. For example, one study found lower live birth rates, clinical pregnancy rates (presence of gestational sacs confirmed by ultrasound), and mean implantation rates when embryos from healthy donors less than 35 years of age were transferred into obese surrogates (BMI≥35) than into lean surrogates (BMI<35) [7]. Other studies have found that obesity affects the window of implantation (WOI) based on gene expression data [8, 9]. Obese women also have elevated levels of advanced glycation end products (AGEs) in uterine lavages and endometrial tissues, suggesting a potential role in the impairment of uterine functions [10]. These data indicate that obesity directly affects the ability of the uterus to support pregnancy, and this increase in pregnancy loss occurs prior to or in the early stages of implantation.

Prior to implantation, embryos are dependent on glucose secreted into the uterine lumen. Too much or too little glucose impairs embryo development. Across species, optimal concentrations of glucose for embryo survival are <4 mM [11, 12]. In agreement, glucose concentrations in the uterine lumen are consistently found to be lower than serum concentrations [13–15]. Thus, the uterine epithelium tightly regulates the amount of glucose entering the uterine lumen. The uterine epithelium also catabolizes glucose for its own metabolic needs, such as proliferation [16, 17]. Our lab has shown that the murine uterine epithelium stores high levels of glycogen at proestrus, which is mobilized during the preimplantation period. The uterine epithelium expresses glucose−6-phosphatase, which is required to secrete glucose released from glycogen [18]. Thus, glucose taken up from maternal circulation or liberated from glycogen can be secreted into the uterine lumen or further metabolized by the uterine epithelium.

An obesogenic environment in humans is characterized by increased intake of calorie-dense foods and reduced physical activity. Typically, it is recommended to consume 20% to 35% of calories from fats. However, studies in humans report that calorie content from fats can reach 30% to 75% [19]. Studies in humans have reported that high dietary fat intake affects reproductive function, specifically leading to an increase in follicle-stimulating hormone, decreased estrogen, and an increase in follicular phase length [3, 20–22]. Overall, the negative impact of obesity on the reproductive outcomes is widely known in humans, yet the mechanism underlying these effects is not fully understood and requires a suitable model. At present, the rodent model is widely used to understand the effect of obesity on reproductive health. While there is no widely agreed-upon fat content to use in DIO studies, 40–60% fat diets are the most commonly used [23–31]. Surprisingly, the effects of diet-induced obesity (DIO) on fertility in mice have been inconsistent, with some finding a decrease in fertility and some finding no effect [23, 31]. For example, one study found that DIO increased the number of implantation sites at day 6, decreased the number of implantation sites at day 9, and had no effect at day 19 in the same experiment [24].

Differences in the outcomes of these studies could be due to several factors. While humans may be obese decades before trying to conceive, the fertility of mice starts decreasing by one year of age [32], limiting the length of DIO studies. Currently, there are no standardized models, and there are multiple varying factors such as diet, the strain of mice, or the timeline of the studies [3, 23–25, 31]. These factors make choosing an appropriate control diet difficult, especially when using multiple high-fat diets in a single study [29, 33]. In many studies focused on fertility, the extent of insulin resistance and metabolic dysfunction is not clear. High-fat diets can also contain significant quantities of phytoestrogens, which could be especially important in reproductive studies [34]. Finally, mouse strains differ in their propensity to develop DIO [35].

Obesity is known to lead to insulin resistance, decreased glycogen stores, and dysregulated glucose metabolism in hepatic and other tissues. Uterine metabolism is important for fertility, but the effects of obesity on metabolism in the endometrium before pregnancy are poorly elucidated [16]. While multiple studies report the negative impact of obesity on decidua [23, 24, 36], we hypothesize that obesity alters glucose metabolism in the uterine epithelium at proestrus, when glycogen levels peak [18].

To achieve this, our first goal was to thoroughly characterize our DIO models. We fed C57BL/6 mice semi-purified diets containing 10%, 45%, or 60% kcal from fat and monitored body mass and glucose intolerance over time. At 18 weeks, mice were sacrificed, and an insulin-resistant phenotype was confirmed by analyzing hepatic glycogen, triglyceride concentrations, and serum leptin concentrations. Finally, we used these mice to assess how DIO affected glycogen metabolism and glycolysis in the uterine epithelium at proestrus. This represents a well-characterized mouse model with clear insulin resistance that can be used in future studies on DIO and fertility.

## Methods

### Experimental models and diets

**Fig. 1:**
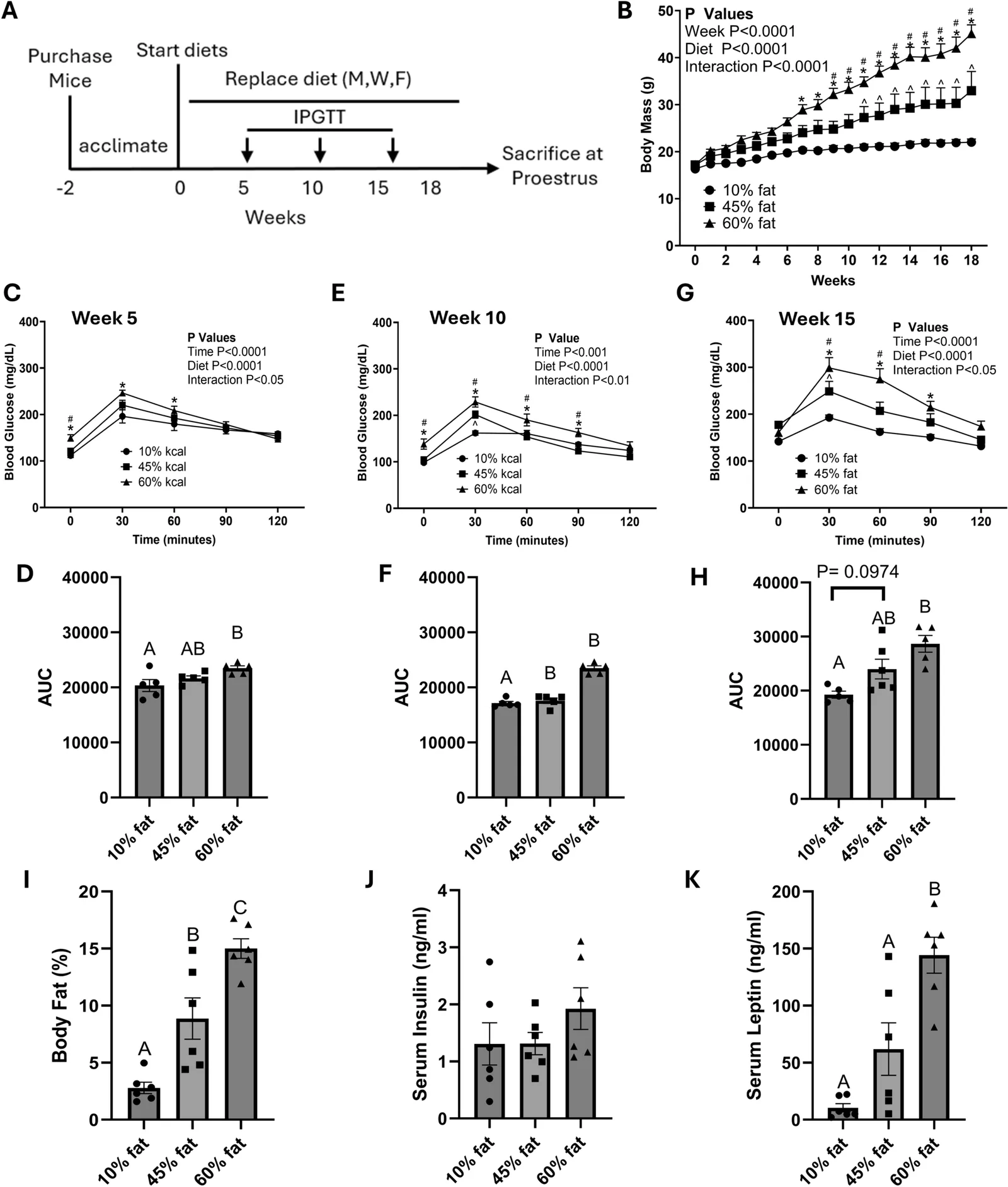
High-fat diets led to an obese phenotype in a dose- and time-dependent manner. **A** Overview of the experimental design. IPGTT, intraperitoneal glucose tolerance test. **B** Body masses from week 0 (8 weeks of age) to week 18 of feeding. **C-H** Glucose levels post-intraperitoneal glucose tolerance test and the respective area under the curve after 5 weeks (**C-D**), 10 weeks (**E–F**), and 15 weeks (**G-H**). **I** Body fat percentage after sacrifice following 18 weeks on each indicated diet. **J-K** Concentration of insulin (**J**), and leptin (**K**) in the serum of mice. *P<0.05 for 60% fat compared to 10% fat. ^*P*<0.05 for 45% fat compared to 10% fat. ^#^*P*<0.05 for 60% fat compared to 45% fat. ^A−C^Bars without the same letter differ, *P*<0.05. n=4–6

Eighteen six-week-old C57BL/6 mice were purchased from Charles River Laboratories (strain #027). Mice were housed 4 per cage and given 2 weeks to acclimate to the animal facility on standard rodent chow containing 18% kcal from fat (#2918, Teklad Diet, Inotiv). Facilities were maintained on a 12 h light/12 h dark (12L/12D) cycle. Mice (n= 6/group) were then randomly assigned to one of three experimental diets (week 0). Semi-purified experimental diets (10% fat [#D12450J], 45% fat [#D08091803], and 60% fat [#D12492]) were purchased from Research Diets Inc. The formulations of the three diets selected were comparable with changes in the percentage kcal from carbohydrates and fats. However, the percentage kcal from sucrose remained the same in all three diets. Also, the phytoestrogens were undetectable in the diets (personal communication with the Research Diets). The 10% and 60% diets were isocaloric (4037 kcal), while the 45% diet was nearly isocaloric (4057 kcal). The complete formulations for all diets are provided in Supplemental Table 1 (Supplementary file 5). Additionally, in an ongoing study, we have observed that the 60% fat diet reduces fertility (unpublished observation). Diets were replaced 3× per week (M, W, and F) to avoid fat oxidation and provided ad libitum (Fig. 1A). All animal procedures were approved by the University of Illinois Institutional Animal Care and Use Committee.

### Intraperitoneal glucose tolerance tests

At the end of the 5th, 10th, and 15th weeks of feeding, an intraperitoneal glucose tolerance test (IPGTT) was performed. Animals were transferred to a new cage and fasted overnight (8–12 h) with access to water. Glucose (1 g/kg body weight) was administered intraperitoneally. A drop of whole blood was taken from the tail vein and glucose was measured using an Ascensia CONTOUR NEXT blood glucose monitor (Contour Next EZ, Ascensia Diabetes Care US Inc, NJ, USA) at 0, 30, 60, 90, and 120 min to measure glucose concentration [37, 38]. A graph was plotted for blood glucose levels, and the area under the curve (AUC) was calculated using GraphPad Prism.

### Estrous cycle

Estrous cycles were monitored by taking daily vaginal lavages starting from the 16th week of feeding for two weeks [18, 29, 39]. Vaginal lavages were collected, placed on a microscope slide, and observed at 10× with a light microscope. The estrous cycle stage was determined based on the abundance of leukocytes, nucleated epithelial cells, and cornified epithelial cells [40]. The mean cycle length was calculated by dividing the total number of days by the number of cycles.

### Animal sacrifice and tissue collection

After 18 weeks, mice were fasted for 5–6 h and sacrificed at proestrus. Mice were euthanized by CO_2_ asphyxiation, and blood was collected via a cardiac puncture. Microfuge tubes containing blood were kept undisturbed for approximately 2 h, and the serum was separated by centrifuging blood at 1,150 × g for 10 min. The serum was collected and stored at − 80 °C. Liver, uterus, abdominal fat, retroperitoneal fat, and gonadal fat were collected and weighed. Tissues were either fixed in 4% paraformaldehyde (PFA) or were snap-frozen and stored at − 80 °C.

### Calculating body fat percentage

Body fat percentage was calculated by dividing the total adipose tissue collected (sum of abdominal, retroperitoneal, and gonadal fat mass) by the body mass of the respective animal × 100. Body fat percentage = (total adipose mass (g)/total body mass (g)) × 100.

### Hematoxylin and eosin staining

The protocol for hematoxylin and eosin staining for adipose was adapted from Berry et al*.* [41]. Adipose tissue and uterine sections (5 µm) were deparaffinized in xylene, immersed in 100% ethanol, and dipped in 95% ethanol for 15 s. The last rehydration step included dipping slides in distilled water for 15 s. The slides were then immersed in hematoxylin (H-3401-500, Vector Laboratories) for 5 min and rinsed under running distilled water till the water became clear. Slides were then dipped in a differentiating solution (1% HCl in ethanol) 5 times and rinsed under running distilled water for a minute. Further, slides were immersed in ammonium hydroxide buffer for 30 s and rinsed again under running distilled water for a minute. Slides were moved to 70% ethanol for 1 min and incubated in Eosin Y (6,766,007, Thermo Fisher) for 30 s and then added to 95% ethanol for 30 s. Slides were incubated in 100% ethanol for 3 min and dehydrated in xylene for 45 s.

The slides were mounted with Permount mounting medium (SP15100, Fisher Scientific), and a coverslip was added. They were dried overnight. Images were captured using a Zeiss Axioskop Microscope (Zeiss Microscopy) with a 305 Axiocam color camera (Carl Zeiss Microscopy). Adipocyte perimeter was measured using ImageJ software v1.54 (http://imagej.nih.gov/ij, National Institutes of Health). Images of the uterine sections were captured by scanning slides with a NanoZoomer 2.0-HT Slide Scanner (Hamamatsu), and the endometrial thickness was measured.

### Insulin and leptin assays

Fasting serum samples were used to measure the serum insulin and leptin levels using a commercially available ultra-sensitive mouse insulin ELISA kit (90080, Crystal Chem) and a mouse leptin ELISA kit (KMC2281, Invitrogen), respectively. Both assays were performed as per the manufacturer’s protocol. For the insulin assay, 5 µL of serum sample was added to the precoated microplate along with 95 μL sample diluent provided in the kit and incubated at 4 °C for 2 h, followed by a thorough washing of wells. Subsequently, the microplate was incubated for 30 min at room temperature (RT) with 100 µL of anti-insulin enzyme conjugate and washed. Lastly, 100 µL of enzyme substrate solution was added and left to react at RT for 40 min. After adding the stop solution, the plate was read at 450 nm and 630 nm. A standard curve was used to determine the concentration of insulin in samples.

In the case of the leptin ELISA, samples were diluted 20-fold. Like with the insulin assay, diluted serum (100 μL) was added to an antibody-coated plate and incubated for 2 h at 37 °C. Samples were aspirated, washed thoroughly, and incubated with 100 µL of leptin biotin conjugate for an hour at RT. Further, 100 µL Streptavidin-HRP was added to the plate and incubated for 30 min at RT before washing the plate. Finally, the plate was incubated in the dark with 100 µL of stabilized chromogen for 30 min at RT and read at 450 nm after adding 100 µL of stop solution. Leptin concentrations in samples were calculated based on the standard curve.

### Glycogen assay

Glycogen content in the liver was measured using a modified assay that we have previously used for cells [42, 43]. The tissue was thawed, and approximately 15 mg of tissue was weighed and homogenized in 400 µL of 30% potassium hydroxide on ice. The homogenate was centrifuged at 18,000 × g at 4 °C for 20 min, and the supernatant was collected. Standards and samples were heated at 95 °C for 30 min and briefly centrifuged. Standard and sample tubes were centrifuged at 1,500 × g for 20 min after adding 480 µL of 100% ethanol. The supernatant was decanted, followed by washing the pellet in 250 µL of 70% ethanol and centrifuging it again. The supernatant was decanted, and the pellet was left to dry overnight. The next day, 50 µL of 1 M HCl was added to the tubes and heated at 95 °C for 3 h to hydrolyze glycogen into glucose. After briefly centrifuging the standards and samples, an equal volume of 1 M NaOH was added to these tubes to neutralize the acidity. In a 96-well plate, 40 µL of standard and sample were added and incubated with 250 µL of Wako reagent (997-03001, Fujifilms) for 30 min at 37 °C. Absorbance was read at 505 nm. Absorbance was compared to the standard curve to determine the amount of glycogen. The amount of glycogen was normalized to the mass of the tissue used for each mouse, and glycogen was expressed as µg of glycogen per mg of liver tissue.

### Periodic Acid–Schiff staining

Periodic Acid–Schiff (PAS) staining was used to histologically quantify glycogen, as we have described before [18, 44]. Liver and uterine sections (5 µm) were deparaffinized in xylene (534056, Sigma-Aldrich) twice for 10 min, followed by rehydration in an ethanol gradient (100%, 95%, 70%, 50%) for 3 min each. Two slides for each mouse were used. One slide was incubated in phosphate-buffered saline (PBS), while the other slide was incubated in 0.5% diastase (PAS+D; 09962, Sigma-Aldrich) at 37 °C for 60 min to digest glycogen in the case of liver sections, and 5 h at RT for uterine sections. Post incubation, both slides were immersed in 0.5% periodic acid solution (AC453171000, Fisher Scientific) for 5 min at RT. Slides were rinsed in distilled water three times and incubated in Schiff’s reagent (3952016, Sigma-Aldrich) for 15 min at RT. Slides were rinsed under running lukewarm water for 5 min. Slides were then counterstained with hematoxylin for 1 min and dipped in ammonium hydroxide buffer for 20 s. Slides were dehydrated in an ethanol gradient (50%, 70%, 95%, and 100%) for 3 min each and then incubated in xylene overnight. The following day, slides were mounted with Permount (SP15100, Fisher Scientific), a coverslip was added, and dried.

Images were captured by scanning slides with a NanoZoomer 2.0—HT Slide Scanner (Hamamatsu).

Diastase-labile PAS staining, indicative of glycogen, in endometrial tissues, was quantified as we previously described [18]. Images were exported from the NanoZoomer (Hamamatsu) software as JPEGs. Using ImageJ (https://imagej.nih.gov/ij/), the total area occupied by each tissue (stroma, luminal epithelium and glandular epithelium) in each image was determined. Then the area stained with PAS was also measured. The area positive for PAS was normalized as the percent of the total area for each tissue. The percent area in PAS+D slides was subtracted from the corresponding PAS images to calculate diastase-labile PAS. For each tissue, measurements were then normalized to the low-fat (10%) diet.

### Triglyceride assays

Liver tissue was thawed, and approximately 15 mg of liver tissue was added to 500 µL of PBS and homogenized on ice. Homogenate was centrifuged at 18,000 × g at 4 °C for 20 min. The supernatant was collected in a separate tube. Triglyceride concentration in the liver tissue was measured using Infinity™ Triglyceride Liquid Stable Reagent (TR22421, Thermo) as per the manufacturer’s instructions. For quantification, 300 µL of triglyceride reagent was mixed with 3 µL of serum or tissue extract samples and incubated for 5 min at 37 °C in a 96-well plate, and the plate was read at 550 nm. The total amount of triglycerides in the tissue piece was normalized to the mass of the tissue piece, and results are expressed as μg of triglyceride per mg of tissue.

### Non-targeted metabolomics

Serum samples (25 μL) and the uterine tissue (approximately 30 mg) were collected from mice at the end of the 18th week at the proestrus stage and were submitted to the core facility. Untargeted gas chromatography-mass spectrometry (GC–MS) metabolite profiling was performed by the Carver Metabolomics Core, University of Illinois Urbana-Champaign, Roy J. Carver Biotechnology Center, as previously described [45]. Samples were initially spiked with an internal standard (hentriacontanoic acid at 10 mg/mL) prior to processing. Each sample was analyzed using a GC–MS system consisting of an Agilent 7890 gas chromatograph (Agilent Technologies), Agilent 5975 MSD, and HP 7683B autosampler. GC was performed on a ZB- 5MS (60 m × 0.32 mm I.D. and 0.25 μm film thickness) capillary column (Phenomenex). The inlet and MS interface temperature was 250 °C, and the ion source temperature was adjusted to 230 °C.

A 1 μL aliquot was injected with a split ratio of 7:1. The helium carrier gas was held at a flow rate of 2 mL/min. Isothermal heating was set at 70 °C for 5 min, followed by an oven temperature increase of 5 °C/minute to 310 °C, then the final 10 min at 310 °C. The MS was operated in a positive electron ionization (EI) mode at 69.9 eV ionization energy with the scan range at m/z 30–800. Peaks were identified using the Automatic Mass Spectral Deconvolution and Identification System (AMDIS) v2.71 (National Institute of Standards and Technology, MD) software and a custom-built MS database derived from in-house chemical standards and the National Institute of Standards and Technology (NIST) database for annotation confirmation. All data were normalized to the internal standard (hentriacontanoic acid at 10 mg/ml).

The data from non-targeted GC–MS were further analyzed using MetaboAnalyst 6.0 for statistics and pathway enrichment [46]. Metabolite data were filtered prior to analysis for both statistical and pathway enrichment analyses. Metabolites with 50% of missing values were removed from the analysis. Data was normalized by the autoscaling method.

Partial Least Squares Discriminant Analysis (PLS-DA) and One-Way ANOVA were performed for the statistical analysis. Enrichment analysis (i.e., Over Representation Analysis) was performed on all metabolites that differed according to the ANOVA (*P*<0.05). Compound names and KEGG IDs for the metabolite library were used to perform enrichment analysis using KEGG. Other parameters were set to default.

### Immunohistochemistry

Immunohistochemistry was done as previously described [18, 44]. Uterine sections (5 μm) were deparaffinized and rehydrated. Slides were boiled in sodium citrate buffer (Fisher Scientific; S271‐3) and incubated in 3% hydrogen peroxide (Fisher Scientific; H325‐500). Slides were incubated with the blocking solution. Block was made of 10% goat serum (Vector Laboratory; S‐1000‐20) and 5% bovine serum albumin (BSA; Fisher Scientific; BP9706100) in tris-buffered saline (TBS). After the block, primary antibodies (Supplemental Table 2 [Supplementary file 5]) were diluted in the block, added to tissue sections, and incubated at 4 °C overnight in hydrated chambers. Our lab has previously validated primary antibodies for glycogen-metabolizing enzymes [44].

The next day, slides were washed in TBS with 0.1% Tween (TBS-T) three times and incubated with secondary antibody (Vector Laboratories; BA‐5000‐1.5) in the block for 30 min at RT. Slides were washed three times and incubated with avidin–biotin complex reagent (Vector Laboratory; SP‐2001) for 30 min at room temperature. After three washes in TBS‐T, 3,3′‐diaminobenzidine (Vector Laboratory; SK‐4100) was applied until brown staining was observed. For each antibody, the reaction was kept the same. Slides were counterstained with hematoxylin for 2 min. The tissues were then dehydrated, mounted, and imaged with a NanoZoomer 2.0—HT Slide Scanner (Hamamatsu). Negative controls were treated as described above, except that the primary antibody was replaced with an isotype-matched negative control (anti‐green fluorescent protein) antibody. Immunohistochemistry was further quantified using QuPath software [47]. Positive cells were detected in the luminal epithelial cells, glandular epithelial cells, and stroma. The percentage of the positive cells was calculated by dividing the number of positive cells by the total number of cells and multiplying by 100.

### Statistical analysis

Analysis for metabolite profiling was performed using MetaboAnalyst 6.0 [46] as described above. All other statistical analyses were conducted using GraphPad Prism version 10.4.1. Body mass and glucose tolerance were analyzed by Two-Way ANOVA, and Tukey’s multiple comparison test was used to compare treatments within each time point. Additionally, the area under the curve (AUC) was calculated for glucose tolerance and compared with One-Way ANOVA and Tukey’s post hoc analysis.

All other data were tested for outliers with a Grubbs’ Test, and outliers were removed. Due to lower sample size, data was assumed to be normally distributed based on the ANOVA’s assumptions principle [48]. However, the Brown-Forsythe test was applied to determine equal variance in the datasets. If treatments had equal variance, data were analyzed using an ANOVA and Tukey’s post hoc analysis. If treatments had unequal variance, data were analyzed using Welch’s ANOVA followed by a Dunnett’s T3 for multiple comparisons. Data is presented as mean ± SEM. A *P*-value < 0.05 was considered statistically significant. P-values from the Brown-Forsythe test for equal variance and the test used to compare means in each experiment are given in Supplemental Table 3 (Supplementary file 5).

## Results

### High-fat diets result in obesity and glucose intolerance

The outline of the experiment is shown in Fig. 1A. After 6 weeks on the experimental diets, the mice in the 60% fat group had a significantly higher body mass than the 10% fat group. At 11 weeks, the body mass of all three groups differed significantly from one another, and the differences continued to increase (Fig. 1B). We also sought to monitor the development of glucose intolerance during the experiment using IPGTTs. After 5 weeks, IPGTT showed slight glucose intolerance in the 60% fat group. The area under the curve (AUC) was higher for the 60% group than the 10% group (Fig. 1C-D). After 10 weeks, mice on the 60% fat diet had developed further glucose intolerance. AUC for the 45% and 60% diets was higher than that of the 10% control (Fig. 1E-F). At 15 weeks, the 45% fat diet tended to increase AUC (*P*=0.0974) relative to the 10% diet, and the 60% group had significantly higher AUC (Fig. 1G-H).

After 18 weeks, mice in proestrus were fasted for 5–6 h and sacrificed. Body fat percentage was 2-fold higher in the 45% fat group than in the 10% fat group (*P*<0.05). In the 60% fat mice, the body fat percentage was 4-fold higher than the control group (*P*<0.0001) and 70% higher than in the 45% fat group (Fig. 1I; *P*<0.05). HFDs increased gonadal, retroperitoneal, and abdominal fat stores (Supplemental Figure S1A-C) and adipocyte perimeter (Supplemental Figure S1D-E). Fasting serum insulin concentrations did not differ in any group (Fig. 1J). Whereas leptin concentrations in the 60% fat group were significantly higher compared to both the 45% fat and 10% control groups (Fig. 1K; *P*<0.0001).

### Serum metabolites were altered by high-fat diets

**Fig. 2:**
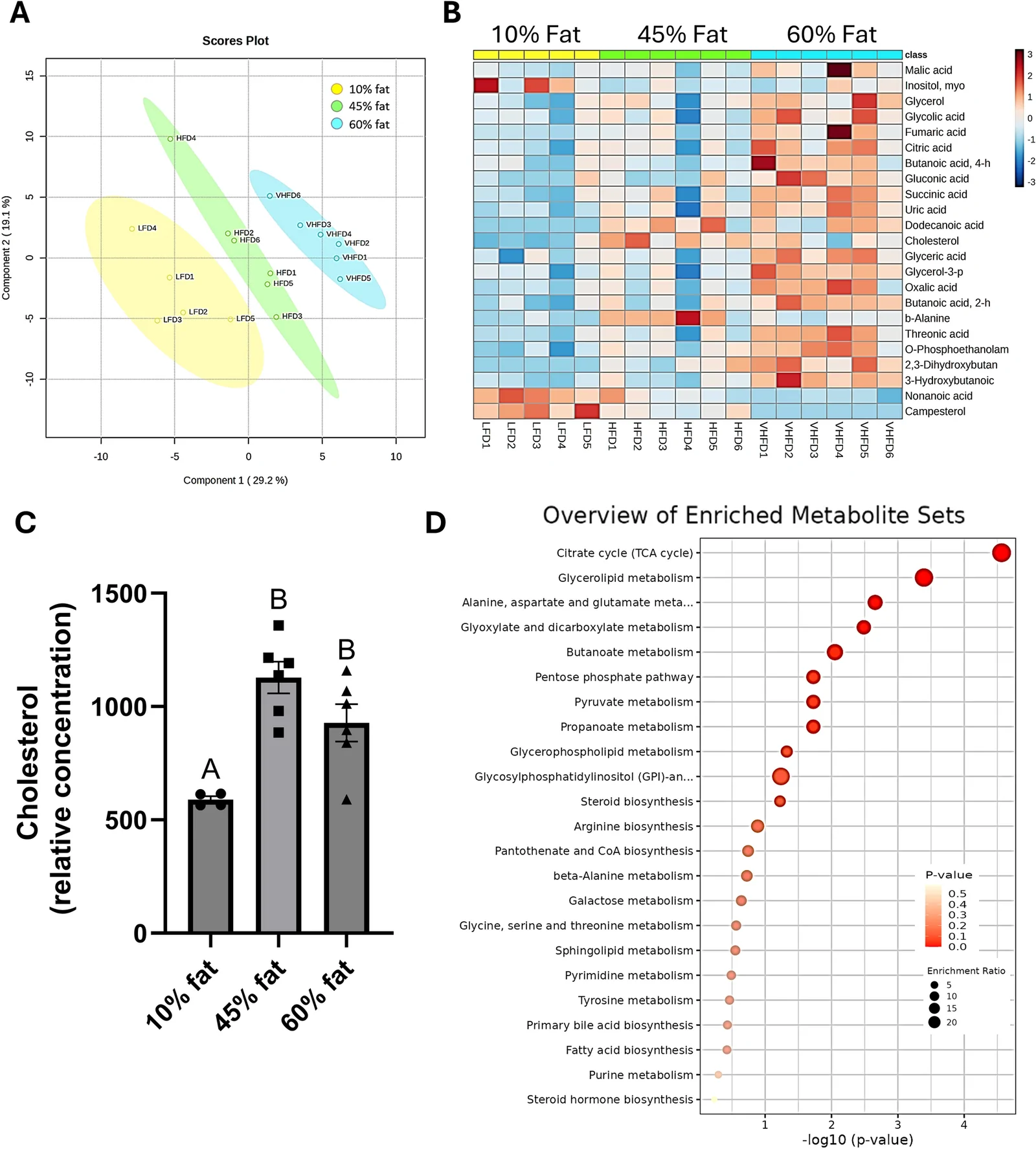
High-fat diets altered serum metabolites. **A** PLS-DA differentiation showing three distinct clusters from all three groups (Yellow- 10% fat group [LFD], Green- 45% fat group [HFD], and Blue−60% fat group [VHFD]). **B** Heatmap of the 23 metabolites that were significantly (*P*< 0.05). **C** Relative concentration of serum cholesterol from all three groups. **D** The top 23 enriched pathways obtained from 23 significantly altered metabolites. n=4–6

To thoroughly characterize changes in serum metabolites due to diet, we performed non-targeted GC–MS on serum. A total of 104 metabolites were detected in at least one sample, but only 86 metabolites were detected in all samples (Supplementary File 1). Three distinct clusters were observed in the PLS-DA analysis without any overlaps (Fig. 2A). Statistical analysis revealed that 23 metabolites were significantly altered (*P*<0.05) as represented in the heatmap in Fig. 2B. Notably, serum from both the 45% fat and 60% fat mice had significantly increased cholesterol levels compared with 10% fat mice (Fig. 2C; *P*<0.05).

Pathway enrichment analysis found 9 pathways were statistically significant with an unadjusted *P*-value < 0.05 (Fig. 2D). These pathways belonged mainly to glucose and energy metabolism (citrate cycle [TCA cycle], the pentose phosphate pathway, pyruvate metabolism, and glyoxylate and dicarboxylate metabolism), lipid and fatty acid metabolism (glycerolipid metabolism, butanoate metabolism, propanoate metabolism, and glycerophospholipid metabolism), and amino acid metabolism (alanine, aspartate, and glutamate metabolism).

### High-fat diets altered the liver metabolism in mice

**Fig. 3:**
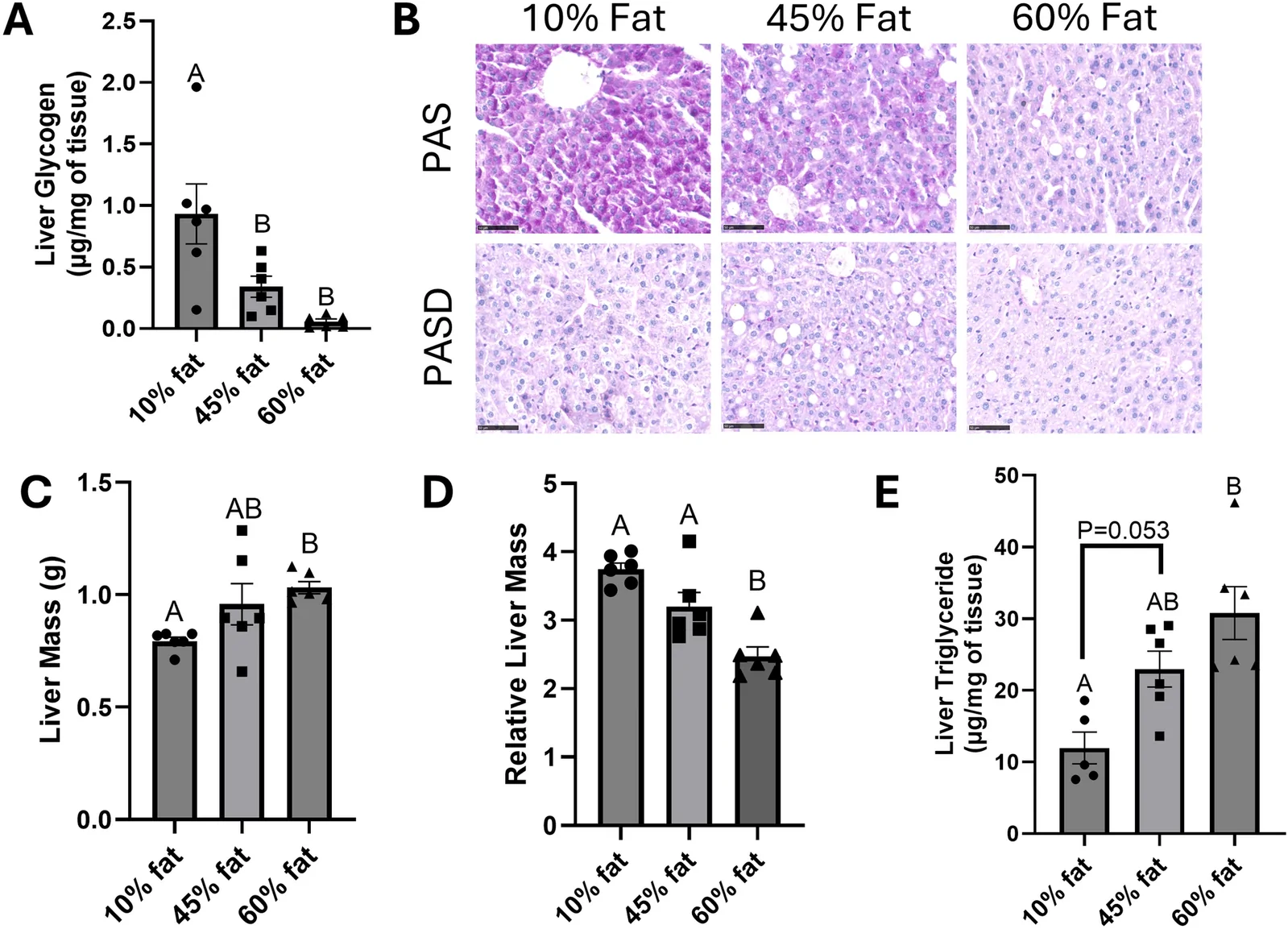
High-fat diets altered liver metabolism relative to the low-fat diet. **A** Glycogen concentrations in the liver collected from mice fed with experimental diets. **B** Periodic acid–Schiff (PAS) and PAS + diastase (PAS+D) staining of liver. Scale bar = 50 μm. **C-D** Liver mass (**C**), and relative liver mass (**D**) in mice fed 10%, 45%, or 60% for 18 weeks. **E** Triglyceride levels in hepatic tissue from mice. ^A−B^Bars without the same letter differ, *P*<0.05. n=3–6

We were interested in seeing how obesity affected uterine glucose metabolism. However, we first wanted to confirm that the systemic effects we observed affected other tissues. Analysis showed that glycogen concentration in the liver was highest in the 10% fat group and significantly lower in both the 45% and 60% fat groups (Fig. 3A-B). In agreement, the 60% diet also increased liver mass, but liver mass relative to body mass was significantly reduced in the 60% fat group (Fig. 3C-D). Hepatic triglyceride concentrations were significantly higher in the 60% fat group relative to the 10% fat group (Fig. 3E). Collectively, these results show that mice on the HFDs developed systemic and hepatic metabolic dysfunction like that seen in obese women, confirming they are a valid model to study the effects of DIO on the uterus.

### Effects of DIO on the reproductive system

We next turned our attention to the reproductive system. The estrous cycles of mice were monitored for 2–3 cycles before being sacrificed at proestrus. Neither estrous cycle length nor the days spent in diestrus or estrus differed among the groups (Supplemental Figure S2A-C). Similarly, the mass of the uterus was not affected, but uterine mass relative to body mass was significantly lower in both high-fat diet groups compared to 10% fat group (Supplemental Figure S2D-E). However, the endometrial thickness was not affected by diet (Supplemental Figure S2F).

### Effects of DIO on uterine metabolism

**Fig. 4:**
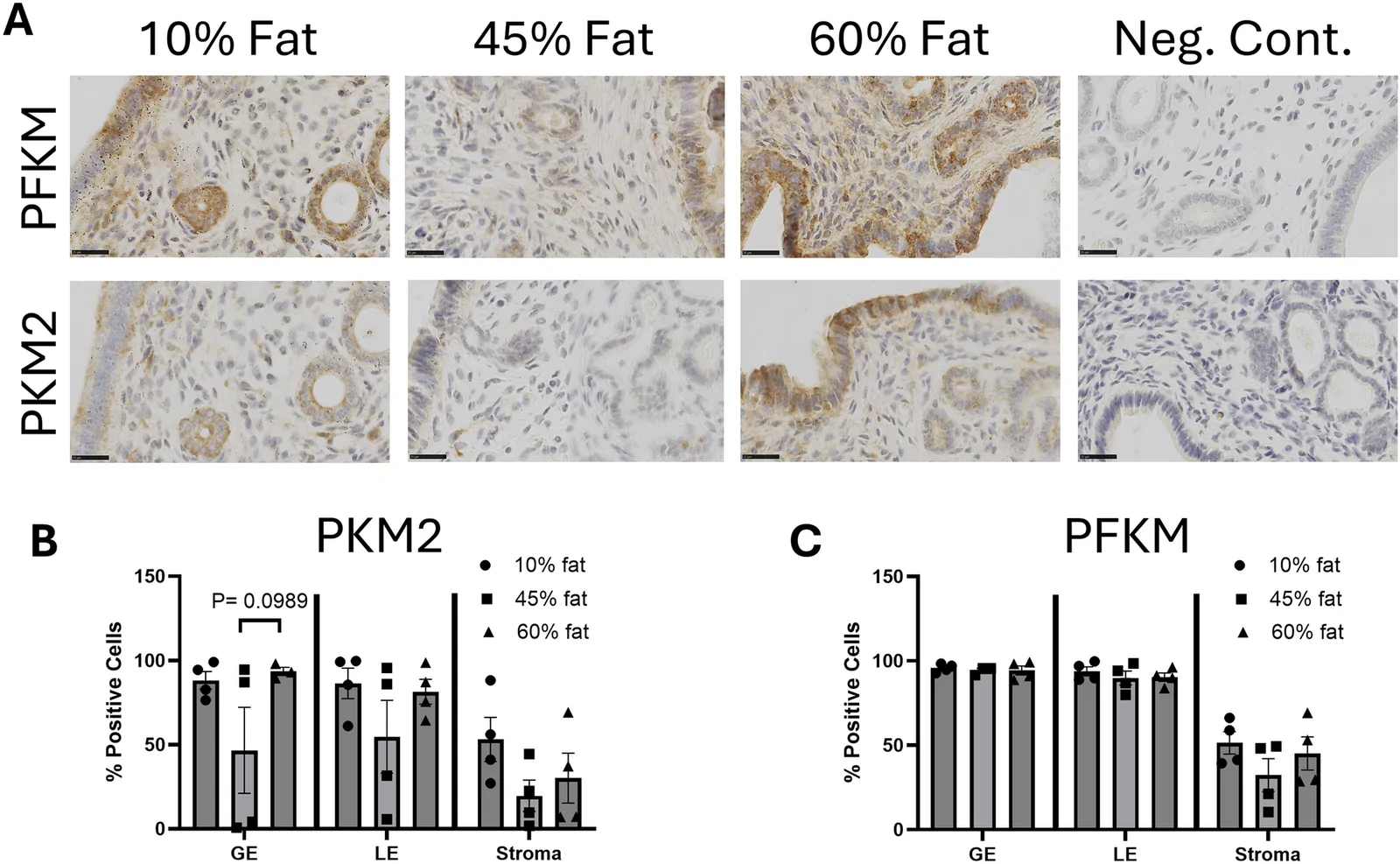
High-fat diets did not affect glucose metabolism. **A** Representative immunohistochemistry images for pyruvate kinase M2 (PKM2) and phosphofructokinase (PFKM) in mice fed the indicated diets for 18 weeks and sacrificed at proestrus. **B-C** Quantified percent positive cells of PKM2 (**B**), and PFKM (**C**) in glandular epithelium (GE), luminal epithelium (LE), and stroma. Neg. Cont. Isotype-matched GFP antibody. Scale bar = 25 µm. n=3–4

Glycolysis is a key pathway to provide ATP for cells, leading us to evaluate the expression of the rate-limiting glycolytic enzymes, phosphofructokinase (PFKM), and pyruvate kinase (PKM2) in the endometrium (Fig. 4A). Immunostaining showed that both enzymes were highly expressed in the glandular epithelium (GE) and luminal epithelium (LE). Immunostaining was detectable in the stroma, but lower than in the GE and LE. Quantification of the immunostaining indicated that, within each tissue, diet had no effect on the levels of PFKM or PKM2 (Fig. 4B-C).

**Fig. 5:**
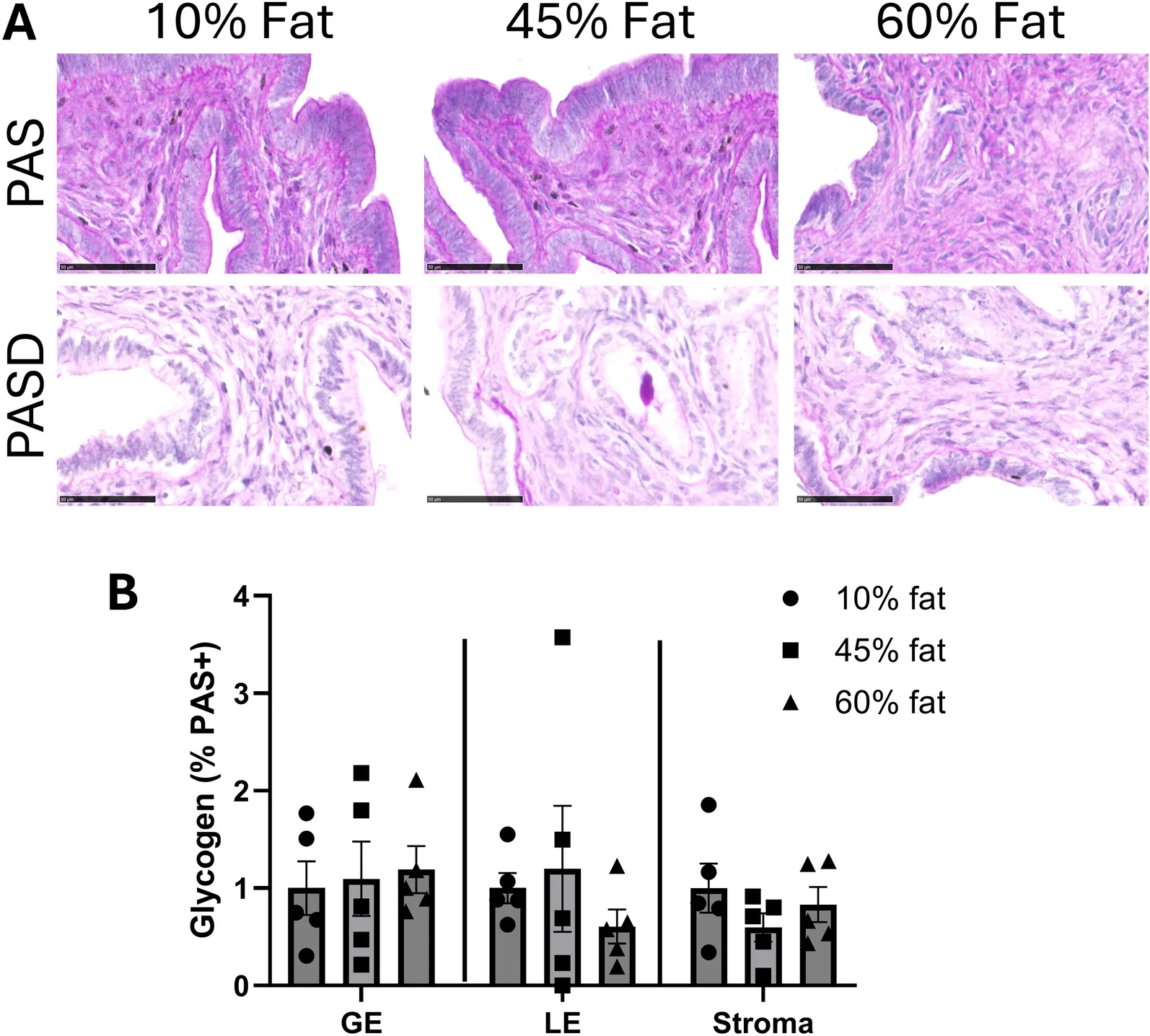
High-fat diets did not affect the glycogen content of the endometrium. **A** Representative images of Periodic acid–Schiff (PAS) and PAS + diastase (PAS+D) from the uterine endometrium of mice. **B** Quantification of diastase-labile PAS staining (indicative of glycogen) in the glandular epithelium (GE), luminal epithelium (LE), and stroma. Scale bar = 50 µm. n=5

**Fig. 6:**
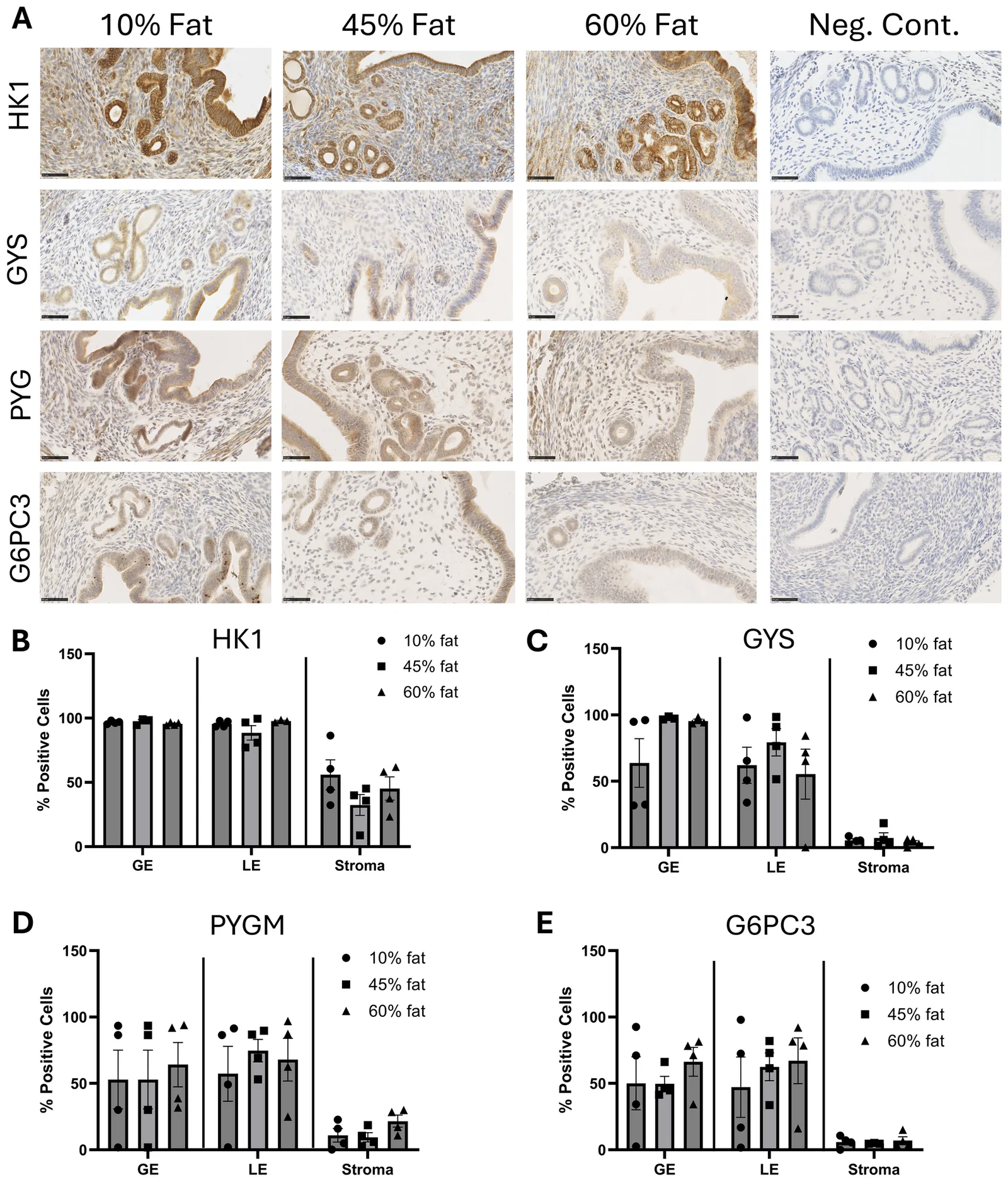
High-fat diets did not affect immunostaining for glycogen-metabolizing enzymes. **A** Representative immunohistochemistry images for hexokinase 1 (HK1), glycogen synthase (GYS), glycogen phosphorylase (PYG), and glucose−6-phosphatase (G6PC3). **B-E** Quantified percent positive cells of HK1 (**B**), GYS (**C**), PYGM (**D**), and G6PC3 (**E**) in glandular epithelium (GE), luminal epithelium (LE), and stroma. Neg. Cont. Isotype-matched GFP antibody. Scale bar = 50 µm. n=3–4

We have shown that the uterine epithelium stores high levels of glycogen at proestrus, which is catabolized during the preimplantation period [18]. Therefore, we determined if DIO affects glycogen levels in the endometrium. In contrast to hepatic glycogen levels, glycogen levels in the GE, LE, and stroma were similar among all three diets (Fig. 5A-B). We also performed immunohistochemistry for the glycogen-metabolizing enzymes hexokinase 1 (HK1), glycogen synthase (GYS), glycogen phosphorylase (PYG), and glucose−6-phosphatase (G6PC3). All four enzymes were highly expressed in the GE and LE, with lower immunostaining in the stroma, agreeing with our previous work [18]. However, there was no difference in immunostaining levels within each tissue for any of the enzymes among the three treatment groups upon quantification (Fig. 6A-E).

### IRS/AKT signaling is unaffected by DIO

**Fig. 7:**
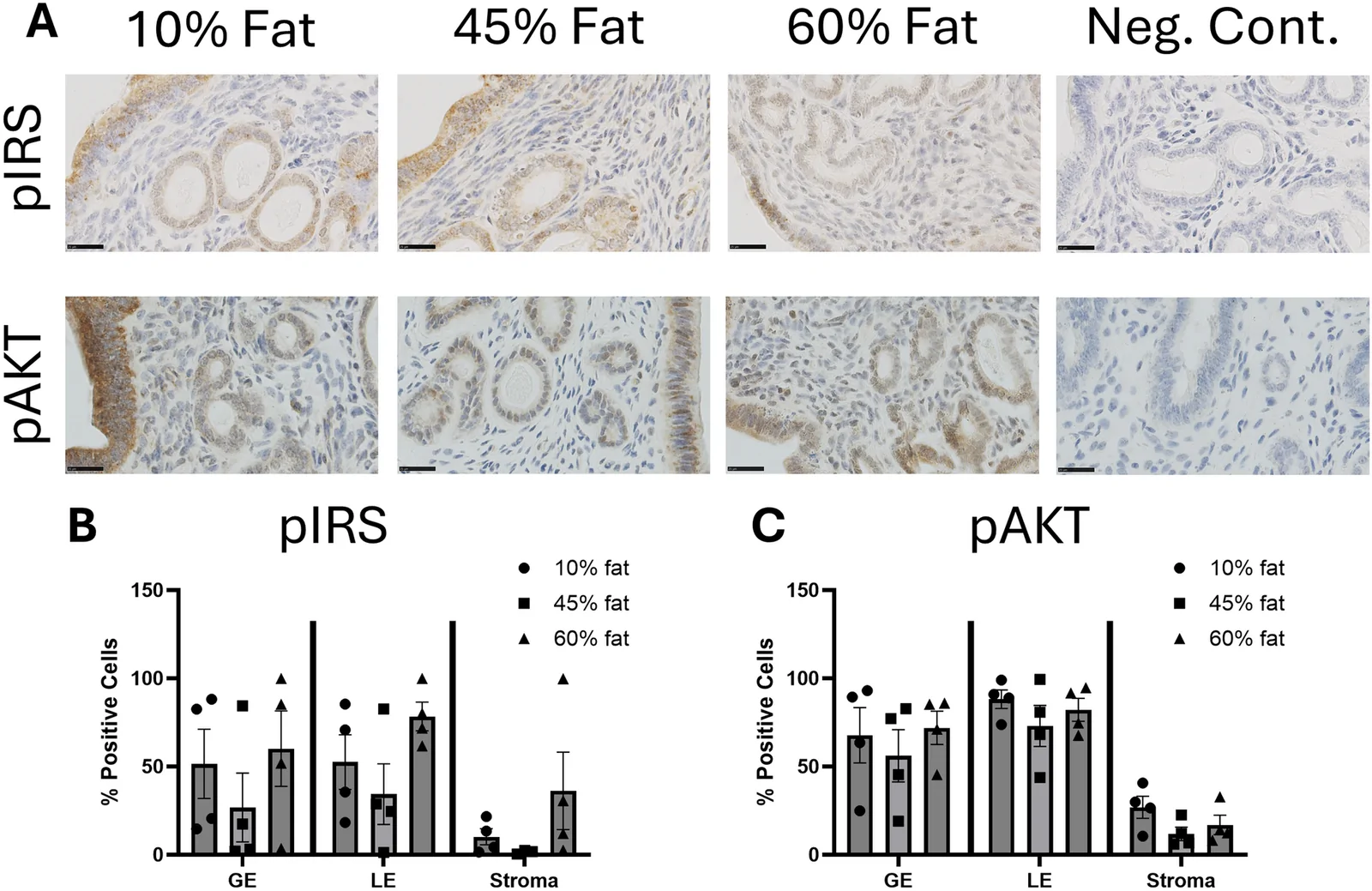
High-fat diets did not affect pIRS or pAKT levels. **A** Representative immunohistochemistry images for phospho-IRS (pIRS), and phospho-AKT (pAKT) in mice. **B-C** Quantified percent positive cells of pIRS (**B**) and pAKT (**C**) in glandular epithelium (GE), luminal epithelium (LE), and stroma. Neg. Cont. Isotype-matched GFP antibody. Scale bar = 25 µm. n=3–4

Glucose metabolism is heavily regulated by IRS/AKT signaling. In the liver, insulin stimulates phosphorylation of IRS and AKT, resulting in increased glycogen levels. Therefore, we wanted to directly examine the effect of DIO on AKT signaling by measuring phosphorylated IRS (pIRS) and phosphorylated AKT (pAKT) levels in the endometrium. Immunohistochemistry showed that diet did not alter the levels of pIRS or pAKT in the GE, LE, and stroma across all 3 diets, confirming that the DIO did not affect IRS/AKT signaling during the course of our experiment (Fig. 7A-C).

### Effect of DIO on uterine metabolites

**Fig. 8:**
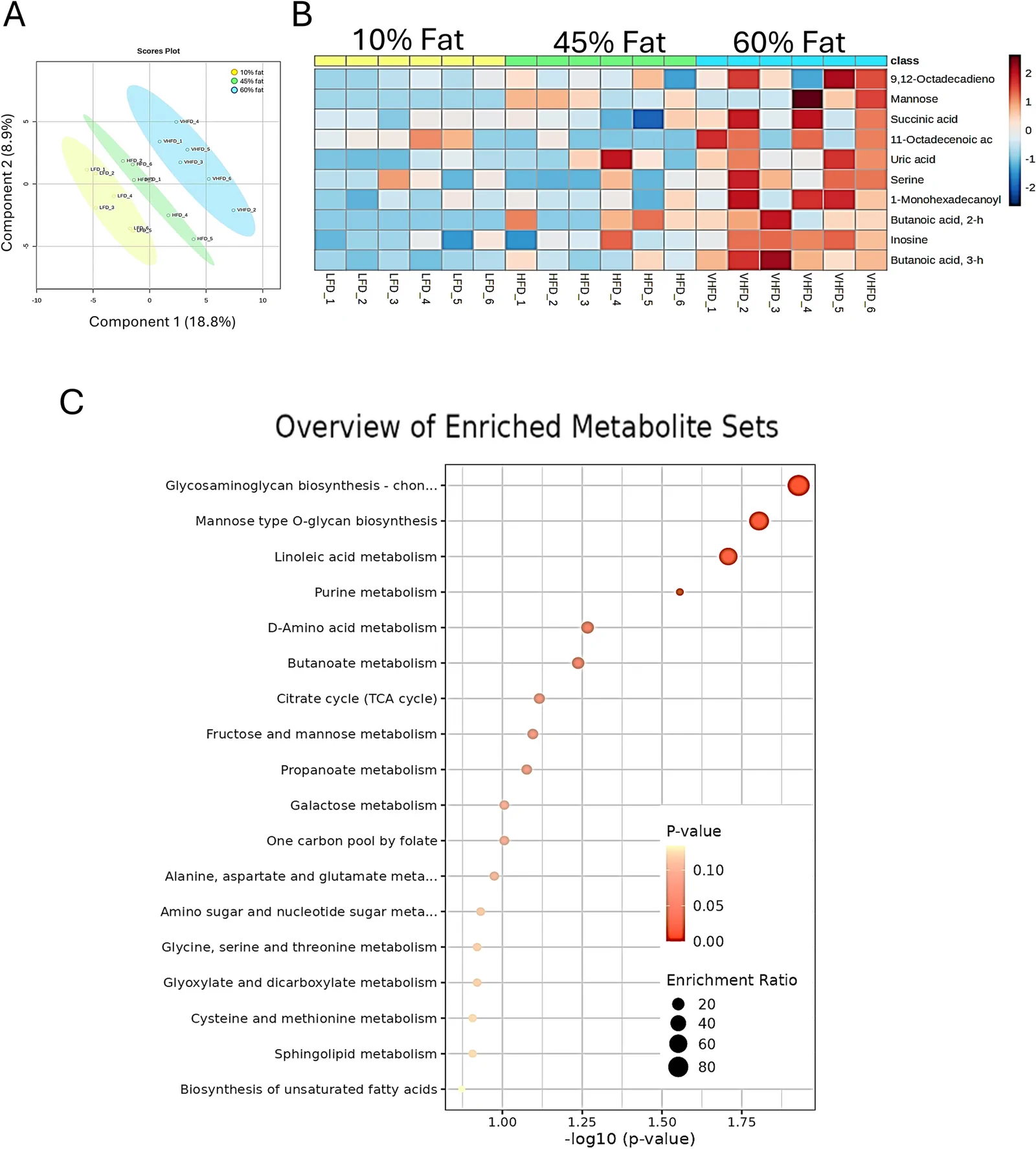
Metabolic changes in the 60% fat group. **A** PLS-DA plot showing three clusters (Yellow- 10% fat group [LFD], Green- 45% fat group [HFD], and Blue−60% fat group [VHFD]). **B** Relative abundance heatmap of 10 metabolites that were significantly altered with P< 0.05. **C** Overview of the top 18 enriched pathways obtained for 10 significantly altered metabolites. n=4–6

Because glucose metabolism and insulin signaling remained unaffected based on what we had measured, we sought a global view of how DIO altered uterine metabolism. Therefore, we performed non-targeted metabolomics (GC–MS). Metabolomic analysis of the uterus at proestrus identified 109 metabolites in at least one sample (Additional File 2). PLS-DA analysis showed distinct clusters for each group, with no overlap of ellipses (Fig. 8A). Based on unadjusted P-values, 10 metabolites differed significantly (Supplementary File 2). A heatmap of relative abundance showed that 10 metabolites were altered primarily in the 60% fat group (Fig. 8B).

Using the 10 metabolites altered by diets (*P*<0.05), a total of 4 pathways were significantly enriched (glycosaminoglycan biosynthesis, mannose type O-glycan biosynthesis, linoleic acid metabolism, and purine metabolism; *P*<0.05; Fig. 8C).

## Discussion

Obesity is a metabolic disease linked to impaired fertility. Women receiving embryos produced using other healthy women’s ova (BMI <24.9 kg/m^2^) consistently find that the BMI of the recipient is negatively associated with pregnancy success [6, 49]. However, fertility results with DIO studies in mice show inconsistencies. Therefore, to unravel the effects of obesity on the uterus before pregnancy, well-characterized mouse models of DIO are needed. Here, we compared the effects of three diets (10%, 45%, and 60%), differing only in the percentage of kilocalories from fat and carbohydrates for a prolonged period of 18 weeks. We thoroughly characterized the systemic effects of our high-fat diets on hallmarks of obesity such as adiposity, hyperleptinemia, and insulin resistance. In an ongoing study on implantation sites, we found infertility increased as the percentage of fat increased, with 70% of mice infertile in the 60% fat group (unpublished observations). These models recapitulated many aspects of human obesity to varying degrees and can be used in future studies to examine how obesity affects the uterus during pregnancy.

Most studies on uterine function in DIO mouse models have focused on the decidua, including studies examining uterine metabolism [23, 24, 50]. While decidualization results in dramatic changes in cell metabolism, metabolism in the uterine epithelium also plays an important role in fertility. For example, glucose provides energy to support epithelial proliferation, which peaks at estrus [17]. Glucose is used to glycosylate proteins in the epithelium, which are required for attachment of the embryo to the epithelium at implantation [51, 52]. Our lab is particularly interested in the regulation of glycogen in the uterine epithelium. We have shown that the glycogen content of the uterine epithelium peaks at proestrus and decreases during the preimplantation period [18]. Thus, epithelial glycogen could sequester glucose in the uterine epithelium prior to mating and regulate intracellular glucose availability during pregnancy.

Obesity is known to affect glycogen metabolism in other tissues, such as the liver. Given the high prevalence of obesity, a logical step was to determine if obesity impairs the ability of the endometrium to store glycogen. The uterine epithelium secretes glucose into the uterine lumen to support the embryo [53, 54]. In our current study, our DIO model experienced decreased glucose tolerance, higher serum leptin concentrations, increased adipocyte size, altered serum metabolites, reduced hepatic glycogen stores, and increased hepatic triglyceride levels. These are all phenotypes observed in obese women and agree with other models of DIO. Interestingly, we did not observe higher fasting concentrations of insulin even though we did observe evidence of insulin resistance.

The mechanisms behind insulin resistance are complex and not yet understood [55]. It is unclear exactly why the endometrium did not become insulin resistant. One reason that the endometrium did not develop insulin resistance could be that insulin may not be a major regulator of uterine metabolism. In vitro, we and others have shown that insulin alters glucose metabolism in the uterine epithelial cells [56, 57, 58]. However, in vivo*,* the evidence is less convincing. Studies from our lab and others have shown that the glycogen content of the uterus changes during early pregnancy or across the non-pregnant cycle, suggesting that factors other than insulin regulate glucose and glycogen metabolism [16, 44, 59, 60]. In agreement, an early study in rats found that exogenous insulin did not affect the glycogen content of the uterus [61]. Conditional knockout of the insulin receptor in the uterus produced only a mild reduction in fertility in mice, though that study did not include any metabolic measures [62]. These results suggest that insulin is not a major regulator of uterine metabolism and that insulin has little effect on fertility.

If insulin is not an important regulator of metabolism in the endometrium, that would explain the lack of effect on obesity in the current study. While knockout of the insulin receptor had minimal effects on fertility, the effect of insulin-like growth factor 1 (IGF1) receptor knockout was more dramatic [63]. We have shown that in vitro, IGF1 stimulates glycogen synthesis in uterine epithelial cells to a similar extent as insulin [42, 56]. Thus, IGF1 could regulate glucose metabolism in the uterus, as has been shown for the brain [63]. Further work is needed to determine if IGF1 or other factors mediate the changes in uterine glucose metabolism that are observed during pregnancy [18, 44, 60].

If the endometrium is not insulin-sensitive, that raises the question of how obesity reduces fertility. While this study focused on the uterine epithelium before mating, it is still possible that obesity impairs decidualization or decidual function. Decidualization results in dramatic changes in glucose metabolism [18, 64, 65]. And there is some evidence that obesity impairs glucose metabolism in the decidua [23, 24]. A second possibility is that obesity affects the hypothalamic-pituitary-ovarian (HPO) axis. There has been much research showing that obesity reduces oocyte and embryo quality [66], but that would not explain the low fertility in obese women who receive donated ova [6, 7]. However, normal HPO axis function is still required after ovulation to support pregnancy. For example, obesity is associated with lower progesterone concentrations in women [67]. Hence, future studies need to investigate how obesity could dysregulate HPO function and, hence, impair the ability of the uterus to support pregnancy.

The current study found few effects of DIO on glucose metabolism in the endometrium. However, this does not rule out the possibility that DIO affects uterine metabolism. Here, we focused on glucose metabolism in the epithelium at proestrus. Other papers have found effects of DIO on glucose metabolism in the decidua [23]. DIO could also affect lipid metabolism. A few enriched pathways we found were related to lipid metabolism, and lipid metabolism of the embryo changes as the embryo enters the uterus [68, 69]. But due to limited availability of the tissue and suitable assays, metabolites that showed significant differences were not verified in the current study. Another limitation of the current study is the reliance on mice. Mice are routinely used in reproductive and obesity studies. Humans can be obese for decades before trying to conceive. However, mice have a short lifespan, limiting how long they can be fed a high-fat diet. In the current study, mice were fed the experimental diets for 18 weeks to maximize the length of feeding without compromising fertility due to advanced age [70].

## Conclusions

We developed and characterized the effects of two high-fat diets on systemic metabolism in C57BL/6 mice. We show that the high-fat diets on the systemic level increased adipose storage and decreased hepatic glycogen levels, classic characteristics of insulin resistance. However, the high-fat diets had relatively minimal effects on glucose metabolism in the endometrium at proestrus, indicating that the endometrium does not become insulin-resistant. After fasting, pIRS and pAKT immunostaining remained unaffected, agreeing that insulin is not a primary regulator of AKT signaling and metabolism in the endometrium at proestrus.

## Supplementary Information

Supplementary file 1.

Supplementary file 2.

Supplemental Figure S1: (A-C) Individual fat mass of animals: gonadal fat mass (A), retroperitoneal fat mass (B), and abdominal fat mass (C) of mice sacrificed at proestrus after 18 weeks on the indicated diets. (D) Representative images of gonadal adipose tissue. (E) Perimeter of gonadal adipocytes from mice fed experimental diets. Scale bar = 100 μm.^A-C^Bars without the same letter differ, P<0.05. n=4-6.

Supplemental Figure S2: (A) Estrous cycle length before sacrifice at proestrus. (B-C) Number of days an animal spent in diestrus (B) or estrus (C) each cycle. (D-F) Uterine mass (D), relative uterine mass (E), and endometrial thickness (F) after 18 weeks of feeding in the three groups.^A-B^Bars without the same letter differ, P<0.05. n=4-6.

Supplementary file 5.

## Data Availability

Mass spectrometry data obtained from the Carver Metabolomics Core at the University of Illinois are provided in Supplementary file 1 and 2. All other data is available upon reasonable request.

## Abbreviations

BMI: Body mass index
CDC: Centers for Disease Control and Prevention
WOI: Window of implantation
AGEs: Advanced glycation end products
DIO: Diet-induced obesity
LFD: Low fat diet
HFD/VHFD: High fat diet/Very high fat diet
IPGTT: Intraperitoneal glucose tolerance test
AUC: Area under the curve
PAS(/D): Periodic Acid Schiff (/Diastase)
GC–MS: Gas Chromatography- Mass Spectrometry
PLS-DA: Partial Least Squares Discriminant Analysis
FDR: False Discovery Rate
ANOVA: Analysis of Variance
KEGG: Kyoto Encyclopedia of Genes and Genomes
IHC: Immunohistochemistry
TBS(/T): Tris buffered saline (/Tween)
GE: Glandular epithelium
LE: Luminal epithelium
PFKM: Phosphofructokinase M
PKM2: Pyruvate kinase M2
HK1: Hexokinase 1
GYS: Glycogen synthase
PYG/PYGM: Glycogen phosphorylase M
G6PC3: Glucose−6-phosphatase
pIRS: Phosphorylated insulin receptor substrate
pAKT: Phosphorylated protein kinase B
GFP: Green fluorescent protein
IGF1: Insulin-like growth factor 1
IGF1R: Insulin-like growth factor 1 receptor
