# Supplementary material for: The effects of diet-induced obesity on glucose metabolism in the mouse endometrium
Source: Uterine Res. 2026 Nov 4;1(1):10. doi: 10.1186/s44494-026-00008-8 (PMC13736471; doi:10.1186/s44494-026-00008-8)
Supplement: Supplementary file 1 [file 44494_2026_8_MOESM5_ESM.docx]

**Supplemental Table 1.** Formulation of diets

| Product # | D12450J | | D08091803 | | D12492 | |
| --- | --- | --- | --- | --- | --- | --- |
| Fat % | 10% Fat | | 45% Fat | | 60% Fat | |
|  | gm% | kcal% | gm% | kcal% | gm% | kcal% |
| Protein | 19 | 20 | 24 | 20 | 26 | 20 |
| Carbohydrate | 67 | 70 | 41 | 35 | 26 | 20 |
| Fat | 4 | 10 | 24 | 45 | 35 | 60 |
| kcal/gm | 3.8 |  | 4.7 |  | 5.2 |  |
|  |  |  |  |  |  |  |
| Ingredients | gm | kcal | gm | kcal | gm | Kcal |
| Casein, Lactic, 30 Mesh | 200 | 800 | 200 | 800 | 200 | 800 |
| Cystine, L | 3 | 12 | 3 | 12 | 3 | 12 |
| Starch, Corn | 506.2 | 2025 | 176.8 | 707 | 0.0 | 0 |
| Maltodextrin 10 | 125 | 500 | 100 | 400 | 125 | 500 |
| Sucrose | 72.8 | 291 | 68.8 | 275 | 72.8 | 291 |
| Solka Flocc, FCC200 | 50 | 0 | 50 | 0 | 50 | 0 |
| Soybean Oil | 25 | 225 | 25 | 225 | 25 | 225 |
| Lard | 20 | 180 | 177.5 | 1598 | 245 | 2205 |
| S10026B | 50 | 0 | 45 | 0 | 50 | 0 |
| Choline Bitartrate | 2 | 0 | 2 | 0 | 2 | 0 |
| V10001C | 1 | 4 | 10 | 40 | 1 | 4 |
| Yellow FD&C #5, Alum. Lake 35-42% | 0.04 | 0 | 0.03 | 0 | 0.00 | 0 |
| Blue FD&C #1, Alum. Lake 35-42% | 0.01 | 0 | 0.03 | 0 | 0.05 | 0 |
| Total | 1055.05 | 4037 | 858.15 | 4057 | 773.85 | 4037 |

Complete formation for 3 diets used in the current study.

**Supplemental Table 2.** Antibodies used for immunohistochemistry

| **Antigen** | **Catalog No.** | **Technique** | **Dilution** | **Block** |
| --- | --- | --- | --- | --- |
| Phosphofructose kinase M | ab154804  Abcam | IHC | 1:50 | Goat Serum |
| Pyruvate kinase M2 | 4053 Cell Signaling | IHC | 1:200 | Got Serum |
| Hexokinase 1 | 2024 Cell Signaling | IHC | 1:20 | Goat serum |
| Glycogen synthase | 3886 Cell Signaling | IHC | 1:40 | Goat serum |
| Glycogen phosphorylase | A9392 ABclonal | IHC | 1:100 | Goat serum |
| Glucose-6-phosphatase | PA5-70653 Invitrogen | IHC | 1:50 | Goat serum |
| Phospho-AKT serine/threonine kinase 1 (phospho- Ser473) | ab81283  Abcam | IHC | 1:50 | Goat serum |
| Phospho-Insulin receptor substrate 1  (phospho- Ser307) | NBP1-78078  Novus biologics | IHC | 1:300 | Goat Serum |
| GFP | 2956 Cell Signaling | IHC | variable | Goat serum |

**Supplemental Table 3.** P-values from the Brown-Forsythe test for the variance between the datasets

| Figure Panel | Brown-Forsythe P Value | Analysis |
| --- | --- | --- |
| Figure 1D | 0.2470 | ANOVA and Tukey’s |
| Figure 1F | 0.8170 | ANOVA and Tukey’s |
| Figure 1H | 0.2531 | ANOVA and Tukey’s |
| Figure 1I | 0.0051 | Welch’s ANOVA and Dunnett’s T3 |
| Figure 1J | 0.3144 | ANOVA and Tukey’s |
| Figure 1K | 0.0298 | Welch’s ANOVA and Dunnett’s T3 |
| Figure 2C | 0.1547 | ANOVA and Tukey’s |
| Figure 3A | 0.0904 | ANOVA and Tukey’s |
| Figure 3C | 0.0983 | ANOVA and Tukey’s |
| Figure 3D | 0.6002 | ANOVA and Tukey’s |
| Figure 3E | 0.3785 | ANOVA and Tukey’s |
| Figure 4B (GE) | <0.0001 | Welch’s ANOVA and Dunnett’s T3 |
| Figure 4B (LE) | 0.0166 | Welch’s ANOVA and Dunnett’s T3 |
| Figure 4B (stroma) | 0.6643 | ANOVA and Tukey’s |
| Figure 4C (GE) | 0.0500 | Welch’s ANOVA and Dunnett’s T3 |
| Figure 4C (LE) | 0.3394 | ANOVA and Tukey’s |
| Figure 4C (stroma) | 0.3897 | ANOVA and Tukey’s |
| Figure 5B (GE) | 0.5587 | ANOVA and Tukey’s |
| Figure 5B (LE) | 0.1912 | ANOVA and Tukey’s |
| Figure 7B (stroma) | 0.7703 | ANOVA and Tukey’s |
| Figure 6B (GE) | 0.7597 | ANOVA and Tukey’s |
| Figure 6B (LE) | <0.0001 | Welch’s ANOVA and Dunnett’s T3 |
| Figure 6B (stroma) | 0.6377 | ANOVA and Tukey’s |
| Figure 6C (GE) | <0.0001 | Welch’s ANOVA and Dunnett’s T3 |
| Figure 6C (LE) | 0.8886 | ANOVA and Tukey’s |
| Figure 6C (stroma) | 0.4794 | ANOVA and Tukey’s |
| Figure 6D (GE) | 0.3410 | ANOVA and Tukey’s |
| Figure 6D (LE) | 0.3745 | ANOVA and Tukey’s |
| Figure 6D (stroma) | 0.4122 | ANOVA and Tukey’s |
| Figure 6E (GE) | 0.0991 | ANOVA and Tukey’s |
| Figure 6E (LE) | 0.2842 | ANOVA and Tukey’s |
| Figure 6E (stroma) | 0.3488 | ANOVA and Tukey’s |
| Figure7B (GE) | 0.8220 | ANOVA and Tukey’s |
| Figure 7B (LE) | 0.5042 | ANOVA and Tukey’s |
| Figure 7B (stroma) | 0.2100 | ANOVA and Tukey’s |
| Figure 7C (GE) | 0.5704 | ANOVA and Tukey’s |
| Figure 7C (LE) | 0.2898 | ANOVA and Tukey’s |
| Figure 7C (stroma) | 0.8600 | ANOVA and Tukey’s |
| Figure S1A | <0.0001 | Welch’s ANOVA and Dunnett’s T3 |
| Figure S1B | 0.0225 | Welch’s ANOVA and Dunnett’s T3 |
| Figure S1C | 0.0284 | Welch’s ANOVA and Dunnett’s T3 |
| Figure S1E | 0.0028 | Welch’s ANOVA and Dunnett’s T3 |
| Figure S2A | 0.7411 | ANOVA and Tukey’s |
| Figure S2B | 0.1120 | ANOVA and Tukey’s |
| Figure S2C | 0.5256 | ANOVA and Tukey’s |
| Figure S2D | 0.1049 | ANOVA and Tukey’s |
| Figure S2E | 0.0053 | Welch’s ANOVA and Dunnett’s T3 |
| Figure S2F | 0.5742 | ANOVA and Tukey’s |

P-values from the Brown-Forsythe test were used to determine if the data had equal variance. If the variance was equal (P>0.05), the data were analyzed by ANOVA and Tukey’s post hoc analysis. If the variance was unequal (P<0.05), Welch’s ANOVA and Dunnett’s test adjusted for 3 comparisons were used.
